# Sustainable Extraction, Chemical Profile, Cytotoxic and Antileishmanial Activities In-Vitro of Some *Citrus* Species Leaves Essential Oils

**DOI:** 10.3390/ph15091163

**Published:** 2022-09-19

**Authors:** Salwa Bouabdallah, Kevin Cianfaglione, Myriam Azzouz, Gaber El-Saber Batiha, Afrah Fahad Alkhuriji, Wafa Abdullah I. Al-Megrin, Mossadok Ben-Attia, Omayma A. Eldahshan

**Affiliations:** 1 Environmental Biomonitoring Laboratory LBE (LR01/ES14), Faculty of Sciences Bizerta, Carthage University, Zarzouna 7021, Tunisia; 2 University Lorraine, CNRS, LIEC, F-57000 Metz, France; 3 Department of Mathematics Computer Science, Paris Dauphine University, F-75016 Paris, France; 4 Multiverse Computing 170, 20014 Donostia-San Sebastian, Spain; 5 Department of Pharmacology and Therapeutics, Faculty of Veterinary Medicine, Damanhour University, Damanhour 22511, Egypt; 6 Department of Zoology, College of Science, King Saud University, P.O. Box 2455, Riyadh 11451, Saudi Arabia; 7 Department of Biology, College of Science, Princess Nourah Bint Abdulrahman University, P.O. Box 84428, Riyadh 11671, Saudi Arabia; 8 Pharmacognosy Department, Faculty of Pharmacy, Ain Shams University, Cairo 11566, Egypt

**Keywords:** *C. sinensis*, *C. limon*, *C. clementina*, neral, linalool, limonene, anti-parasitic, green extraction

## Abstract

Anti-leishmanial drugs extracted from natural sources have not been sufficiently explored in the literature. Until now, leishmaniasis treatments have been limited to synthetic and expensive drugs. This study investigated, for the first time, the anti-leishmanial efficacy of essential oils (EOs) from the leaves of *Citrus* species (*C. sinensis*, *C. limon*, and *C. clementina*). Essential oils were extracted from three species by solvent free microwave extraction (SFME); in addition, lemon oil was also isolated by hydro-distillation (HD). These were investigated using gas chromatography coupled with mass spectrometry (GC–MS) and evaluated against *Leishmania* species, namely *Leishmania major* and *Leishmania infantum*, using a mitochondrial tetrazolium test (MTT) assay. The chemical compositions of *Citrus limon* EOs obtained by HD and SFME showed some differences. The identified peaks of *C. limon* (SFME) represented 93.96%, where linalool was the major peak (44.21%), followed by sabinene (14.22%) and ocimene (6.09%). While the hydro-distilled oil of *C. limon* contained geranial (30.08%), limonene (27.09%), and neral (22.87%) in the identified peaks (96.67%). The identified components of *C. clementina* leaves oil (68.54%) showed twenty-six compounds, where the predominant compound was geranial (42.40%), followed by neral (26.79%) and limonene (14.48%). However, 89.82% *C. sinensis* oil was identified, where the major peaks were for neral (27.52%), linalool (25.83%), and geranial (23.44%). HD oil of lemon showed the highest activity against *L. major*, with moderate toxicity on murine macrophage (RAW 264.7) cells, and possessed the best selectivity index on both *Leishmanial* species (SI: 3.68; 6.38), followed by *C. clementina* oil and *C. limon* using SFME (0.9 ± 0.29, 1.03 ± 0.27, and 1.13 ± 0.3), respectively. *C. clementina* oil induced the greatest activity on *Leishmania infantum*, followed by HD lemon and SFME lemon oils (0.32 ± 0.18, 0.52 ± 0.15, and 0.57 ± 0.09, respectively) when compared to Amphotericin B (0.80 ± 0.18 and 0.23 ± 0.13) as a positive control, on both species, respectively. Our study suggests a potent anti-leishmanial activity of lemon oil (HD) on *L. major*, followed by *C. clementina*. With the same potency on *L. infantum* shown by *C. clementina* oil, followed by HD lemon oil. This effect could be attributed to the major compounds of limonene, citral, and neral, as well as the synergistic effect of other different compounds. These observations could be a starting point for the building of new anti-leishmanial drugs from natural origins, and which combine different EOs containing *Citrus* cultivars.

## 1. Introduction

Leishmaniasis is a parasite and a vector-borne disease caused by the *Leishmania* genus. This protozoon is known to be a severe public health concern, resulting in high death rates and morbidity in various Mediterranean, Asian, African, and Latin American areas [1]. This disease affects more than 12 million people globally, in ninety eighty countries (tropical and subtropical) [1]. The disease’s manifestation starts with cutaneous leishmaniasis, which is caused by *L. major*, and visceral leishmaniasis, which is caused by *L. infantum*. These parasites exhibit two well-defined forms. The first form is the flagellated extra-cellular promastigote, and the second is the non-motile intracellular amastigote [2]. In the absence of a candidate vaccine, antimony, which is composed of amphotericin B and miltefosine is the latest chemotherapy. Nonetheless, these chemical drugs have several limitations, including various side effects, toxicity, high cost, relapse, and low resistance [3,4]. Effective drug development becomes an essential requirement to avoid these concerns, particularly in the regions of endemic disease. The identification of bioactive natural compounds is an important research area for scientists to fight protozoal infections and their detrimental effects [5], as well as to discover new alternatives to ordinary chemical drugs [6,7].

Essential oils (EOs) exhibit many biological effects, such as larvicidal, insecticidal, anti-cancer, antiviral, antibiofilm, antibacterial, and antiparasitic effects [8]. *Citrus* plants are famous for their volatile oils. The potential of EOs *Citrus* and their compounds, such as limonene, linalool, and citral, has been reported against many organisms, such as *Staphylococcus aureus*, *Listeria monocytenes*, and *Bacillus cereus* [9,10]. Additionally, the leaf oils of *Citrus* have exhibited antimicrobial potential against many pathogens [11]. *C. clementina* EO, which is rich in monoterpenes, confers powerful anti-infectious properties against bacteria, viruses, and fungi, and also worms and parasites [12,13]. *Citrus reticulata* fruit peel showed a high anti-Leishmania *amazonensis promastigote* activity [14]. On the other hand, *Citrus sinensis* dried leaves and EO of *Citrus limon* also displayed antileishmanial activity.

Various EO extraction methods have been developed since antiquity, starting with conventional technologies such as pressing, hydro distillation, steam distillation, and dry distillation [10,14]. The perfect extraction method should be non-destructive, must provide a high yield, and be time-efficient [15]. Natural product green extraction is considered a new concept to solve the challenges of the 21st century, to protect both the environment and consumers, and increase competition between industries for more economic, ecological, and innovative techniques [16]. Within the green extraction approach, the extract obtained should have the lowest possible impact on the environment (less energy and solvent consumption, etc.), and eventual recycling has to be planned for (co-products, biodegradability, etc.) [17]. Among these developed methods, microwave heating is a new technique being considered. It has been broadly applied in natural product extraction, especially in volatile extract papers [18,19].

According to rigorous research of the literature, there are not any data reported on the optimization of the application of microwaves for extraction of EOs from *Citrus* leaf species.

In the present work, we chemically compared (GC-MS) the EO of *C. sinensis*, *C. limon*, and *C. clementina* using solvent-free microwave extraction (SFME), in addition to that of *C. limon* obtained by hydro-distillation (as an example for comparison between hydro distillation and microwave extraction), to evaluate the antiparasitic activity against *Leishmania major* and *Leishmania infantum* in vitro.

## 2. Results

### 2.1. Extraction Yield

All essential oils have a yellow color and pleasant herbaceous scent, especially HD lemon oil. The average yields (calculated based on the initial plant weight) of essential oils (SFME) from the fresh leaves of *C. limon*, *C. sinensis*, and *C. clementina* (measured in triplicate) were 0.30 ± 0.004% *(w*/*w)*, 0.26% ± 0.007 (*w*/*w*), and 0.24 ± 0.002% (*w*/*w*), respectively; while the yield of hydro distilled lemon leaves was 0.33 ± 0.002 *(w*/*w)*.

### 2.2. Quantitative and Qualitative Analysis

**Figure 1:**
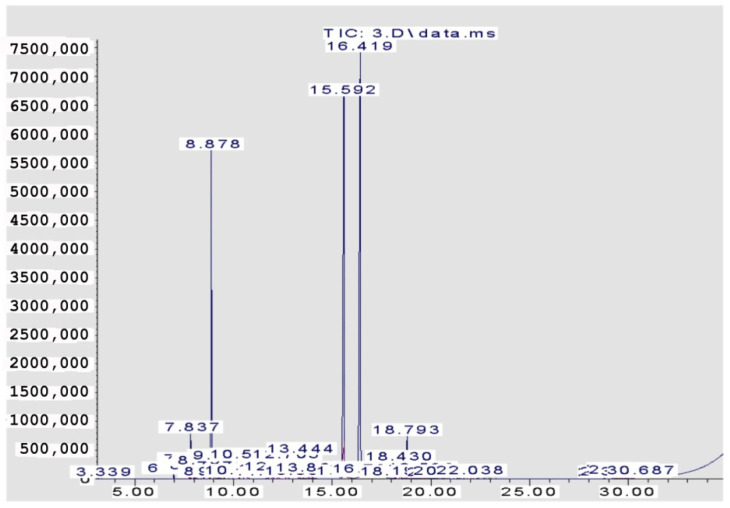
GC Chromatogram of *C. clementina* (SFME).

**Figure 2:**
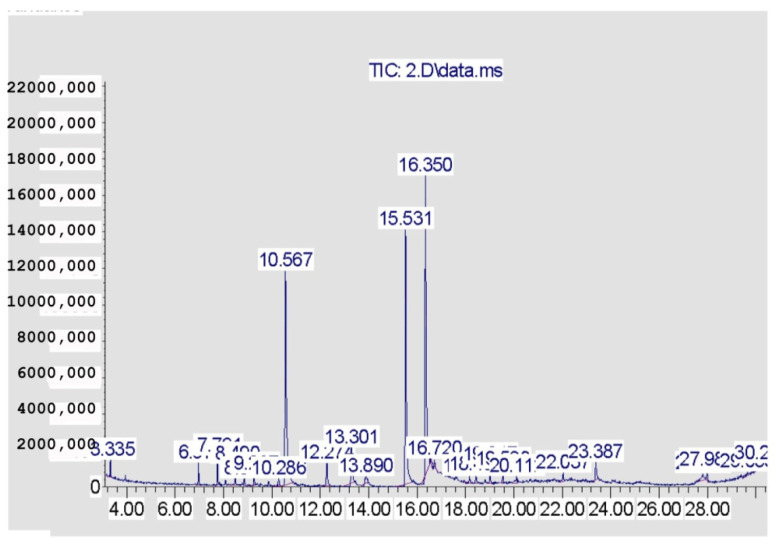
GC chromatogram of *C. sinensis* (SFME).

**Figure 3:**
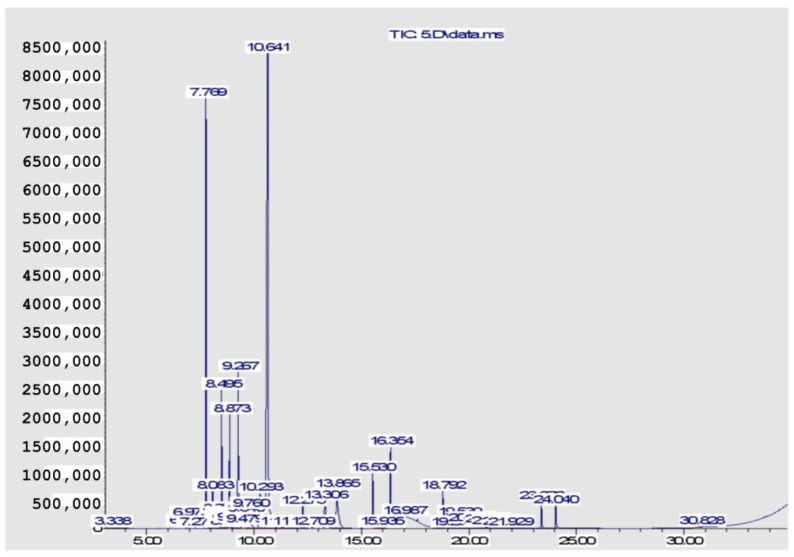
GC chromatogram of *C. limon* (SFME).

**Figure 4:**
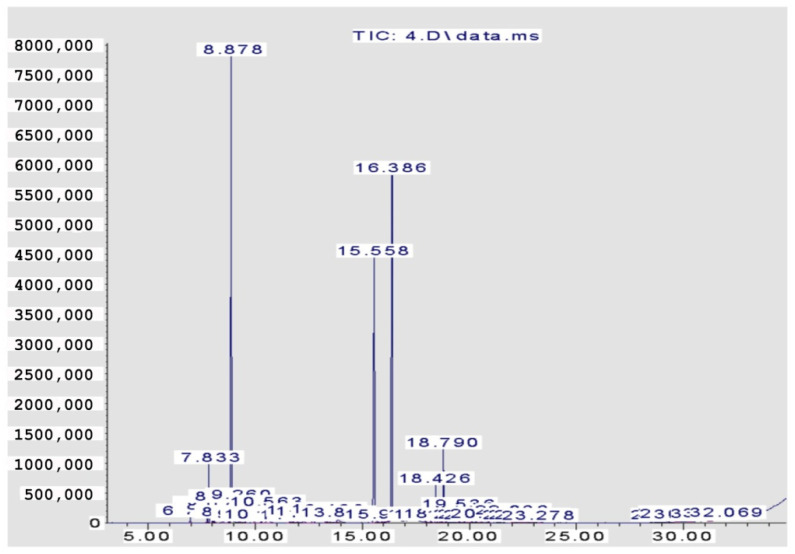
GC chromatogram of *C. limon* (HD).

The volatile components constituting the four EOs from the *Citrus* species are recorded in Table 1 and Figure 1, Figure 2, Figure 3 and Figure 4. A total of 29 compounds were identified in the four samples, accounting for (68.54–96.67%) of the total compositions. Overall, the composition of the EOs was dominated by oxygenated monoterpenes (48.7% in *C. clementina*, 83.12% in *C. sinensis*, 59.99% in *C. limon* obtained by SFME, and 62.12% in *C. limon* obtained by HD) followed by monoterpene hydrocarbons (18.88% in *C. clementina*, 4.37% in *C. sinensis*, 33.35% in *C. limon* obtained by SFME, and 33.64% in *C. limon* obtained by HD). However, the four EOs showed great differences, qualitatively and quantitatively.

Twenty-six components were recognized from the oil of *C. clementina* leaves, representing 68.54% of the total detected components. The major constituents of the oil were geranial (42.40%), followed by neral (26.79%) and limonene (14.48%). However, 89.82% *C. sinensis* oil was identified, where the major peaks were for neral (27.52%), linalool (25.83%), and geranial (23.44%). The peaks identified in *C. limon* represented 93.96%; linalool was the major peak (44.21%), followed by sabinene (14.22%), and ocimene (6.09%). The hydro distilled oil of *C. limon* contained geranial (30.08%), limonene (27.09%), and neral (22.87%) as the predominant compounds in the identified peaks (96.67%).

### 2.3. Anti-Leishmanial Activity

All the EOs, E1 (leaves of *C. limon*; SFME), E2 (leaves of *C. clementina*; SFME), E3 (leaves of *C. sinensis*; SFME), and E4 (leaves of *C. limon*; HD), were tested for their anti-leishmanial activity against both species (Table 2).

**Figure 5:**
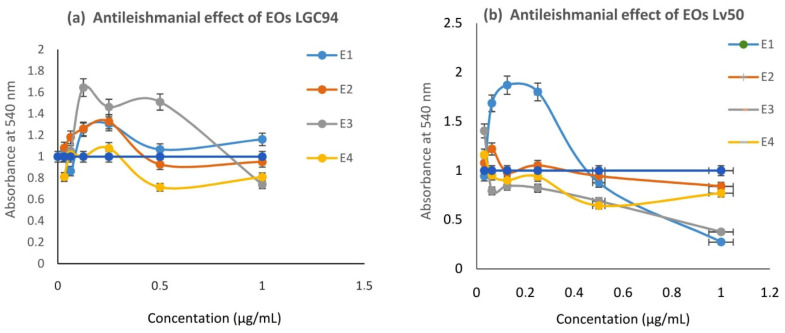
Antileishmanial effect of essential oils (**a**) on GLC94, (**b**) on Lv50. Percentage—add error bars with the default of 5%.

*L*. *infantum* promastigotes were more sensitive than *L*. *major* to all EOs (E1–E4) investigated (Figure 5). The anti-leishmanial activity of the EOs (E1, E2, and E4) was almost the same as amphotericin B. The HD oil of lemon was the most effective against *L. major*, followed by *C. clementina* oil, then *C. limon* from SFME (0.9 ± 0.29, 1.03 ± 0.27, and 1.13 ± 0.3), respectively. *C. clementina* had the greatest effect on *Leishmania infantum*, followed by HD lemon and SFME lemon oils (0.32 ± 0.18, 0.52 ± 0.15, and 0.57 ± 0.09), when compared to Amphotericin B (0.80 ± 0.18; 0.23 ± 0.13) as a positive control on *L. major* and *L. infantum*, respectively. Orange oil showed the weakest effect on both species.

### 2.4. Cytotoxicity

The EOs were also investigated for their potential cytotoxic effect against murine RAW 264.7 cells. The cytotoxicity was determined using the conventional MTT assay, as stated in the methods described by Mossman [20]. All the tested EOs showed cytotoxic activity (IC80%) against RAW 264.7 cells (E2: *C. sinensis* (0.16 ± 0.09) < E3: *C. clementina* (0.18 ± 0.11) < E1: *C. limon*, SFME (0.30 ± 0.39) < E4: *C. limon*, HD (3.32 ± 0.24)) compared to Amphotericin B (9.23 ± 0.13), as shown in Table 2. The HD oil of lemon was the most effective against *L. major*, with moderate toxicity on murine macrophage (RAW 264.7) cells and with the best selectivity index (SI: 3.68; 6.38, respectively; which is between 1 and 10) on both *L. major* and *L. infantum.* Evidently the 1% (*v*/*v*) DMSO used as a solvent for EOs was not cytotoxic to either parasites or the macrophage RAW 264.7 cell line.

## 3. Discussion

*C. sinensis* is an evergreen flowering tree. Its height is from 9 to 10 m. The leaves are alternate, with narrowly winged-petioles, the shape of blades ranges from elliptical, oblong to oval, bluntly toothed and they emit a strong characteristic *Citrus* odor, due to the presence of copious oil [21]. The size of lemon leaves is small to medium. The leaves are oblong, ovate, and taper to a point on the non-stem end. The green vibrant leaves have fine-toothed edges and grow alternately along the branches [22]. Clementine is one of the varieties of *Citrus reticulate* (Mandarin orange). It was named in 1902. The leaves are narrow and small, and look like willow leaf mandarins [23].

### 3.1. Essential Oil Analysis

In comparing our results with those previously reported, it was found that the composition of *C. limon* EOs in Spain, Portugal, Italy, France, Tunisia, and Morocco are characterized by high levels of limonene (56.99–71.87%), myrcene (0.9–1.47%), and geranial (0.1–5.43%), where our results indicated that linalool (44.21%) and geranial (30.08%) are the major compounds in lemon oil (SFME and HD, respectively).

It was reported that the main EO compounds of *C. clementina* in Italy are limonene (95.46%), *α*-pinene (0.43%), and myrcene (1.82%) [24], whereas geranial (42.40%) was the major compound in our sample. The chemical composition of the EO of *C. sinensis* from Canada, Spain, France, Italy, Morocco, and Tunisia was characterized by a very high percentage of limonene exceeding 90%, with myrcene (0.05–3.77%) and linalool (0.04–9%), but in our sample, the major compounds were neral (27.52%) and linalool (25.83%).

In agreement with our GC-MS analysis, Janoti et al. [25] noted that limonene (30.1–47.3%) was the main compound in the leaf oil of certain lemon species that were cultivated in India. Limonene, linalool, and terpineol (36.9%, 2.12%, and 4.19%) were found to a higher extent in *Citrus limettioides* compared to *Citrus* pseudo *limon* (10.6%, 1.7%, and 2.59%).

Our results are also in agreement with those of Vekiari and Aydeoun [26,27], who showed that lemon leaf oil, of various origins, was characterized by high concentrations of limonene. However, our results did not conform to those mentioned by Owolabi [28], as the chemical profile of the oil of lemon leaf in the Owolabi study is poor in neral and geranial (4.5%, 4%).

Both the yield and chemical composition of essential oils differ remarkably, depending on many factors. The qualitative and quantitative variations in the components of oils in this study and others can be attributed to geographic, genetic, and seasonal variations, as well as the organs of plants, ripening stage, cultivar, practices of culture, species, methods of extraction, climate, and environmental conditions [18,24,29].

Interestingly, the EOs from *C. limon* obtained by SFME and HD showed significant differences (Table 1). The former was dominated by linalool (44.21%) and sabinene (14.22%), whereas the latter was characterized by limonene (27.09%), neral (30.08%), and geranial (30.08%). HD extracts showed a higher percentage of monoterpenoids, including respective hydrocarbons and oxygenated (33.64%, 62.12%), than the SFME extracts (33.35%, 59.99%). This result could have been due to the more efficient heat flow induced by the microwaves, which produced a rapid heating step for the whole system; only 1 min during the SFME process but 30 min during the HD process. The whole sample was almost simultaneously heated, which could have resulted in the loss of the most volatile compounds, such as *α*-pinene, *β*-pinene, *β*-myrcene, *δ*-3-carene, and *p*-cymene. The presence of these molecules in the HD extracts explains their characteristic and pleasant floral scent [18]. SFME extracts were characterized by large amounts of alcohols, such as linalool, α-terpineol, geraniol, ketones, and esters. Similarly, Nayak et al. [30] studied the extraction process of *Citrus maxima* (Burm.) using SFME and HD, and if we consider the content of the main compound, SFME was a more effective technique and produced the highest amount of heptenol, borneol, caryophyllene oxide, and *trans*-*β*-farnesene [30]. SFME produced EOs with higher quantities of valuable oxygenated compounds. This may be due to the reduced heating time required, which partially prevents the decomposition of the oxygenated compounds [18,19,24].

EOs are a volatile and semi-volatile mixture of complex of lipophilic compounds, which can be obtained by various techniques, such as pressing, distillation, steam distillation, hydro-distillation, and solvent-free microwave extraction. However, numerous industrial operations have favored using microwaves (MW) for heating fresh materials, noting an exceptional capacity to penetrate at a deep level into materials, since this substantially reduces the processing time. On the other hand, MW heating, compared to the conventional heating process, seems to be a more economical and environmentally friendly technology. Industries that are involved in the extraction of natural products (cosmetics, perfume, food, pharmaceutical, and bio-fuel) have to combine process intensification with safer and cleaner extraction protocols. Process intensification covers all developments of new techniques, equipment, or procedures that bring significant progress in comparison with the current production methods [15,17]. The challenges in the industrial development of intensified processes are multiple: more compact production units and a reduced number of unit operations, raw material savings, energy process safety control, and reduction in waste and ecological footprint [17].

Some studies reported that SFME resulted in a larger yield than the conventional method HD for extracts such as guava (*Psidium guajava*) [31] and lavender (*Lavandulax hybrida*) essential oils [32]. However, other reported data did not show any differences between these methods. Yields of *Ocimum basilicum*, *Rosmarinus officinalis*, *Thymus vulgaris*, and *Mentha crispa* essential oils isolated through SFME and HD were similar quantitatively [31,32]. These results suggest that both SFME and HD can result in a higher yield of EOs, depending on the plant species. However, SFME is more effective than HD, concerning time and energy saving.

### 3.2. Anti-Leishmanial Activity

Regarding our results for EOs isolated from the same lemon cultivar “Eureka”, namely E1 and E4, we noticed that E1 was characterized by linalool: 44.21% and limonene: 4.34%, while E4 was characterized by linalool: 1.59% and limonene: 27.09%. In addition, E2 isolated from the cultivar “Cassar”, containing linalool: 1.68% and limonene 14.48%, exhibited the highest activity on Lv50 (IC_50_ = 0.32 μg/mL). Thus, the biological effect of EOs is probably due to the combined effects of their main components and their minor constituents; the major contributors were linalool, limonene, neral, and geranial. Therefore, we can conclude that limonene, neral, and geranial-containing oils showed antileishmanial activity and that this was achieved in both lemon (HD) and clementine oils (SFME).

Other *Citrus* plants with nearly the same components exerted an antileishmanial activity, as limonene 85.7% was the major compound of *Citrus reticulata* fruit peel [13], while lemon leaf oil contains neral and geranial as major compounds [33].

Limonene possesses low toxicity in humans and could be used as an enhancer for the percutaneous permeation of drugs. Limonene is a monoterpene that has antitumoral, antibiotic, and antiprotozoal activity. Limonene killed both *Leishmania amazonensis* promastigotes and amastigotes (IC50; 252.0 49.0 and 147.0 46.0 mM). It induces activity against *Leishmania braziliensis*, *Leishmania major*, and *Leishmania chagasi* promastigotes. A 300-mM limonene treatment of limonene to *L. amazonensis*-infected macrophages led to a reduction of 78% in infection rates. Topically or intrarectally treating mice with limonene that were infected by *L. amazonensis* resulted in a significant reduction in the sizes of lesions [34]. A linalool-rich essential oil from *Croton cajucara* induced potent antileishmanial activity with 50% lethal doses, 8.7 ng/mL for amastigotes and 8.3 ng/mL for promastigotes, that inhibited the L. amazonensis promastigotes growth at very low concentrations; MIC, 85.0 pg/mL [35]. *Myrcia ovata*, with geranial and neral as major constituents, was highly active, with IC_50_/96 h of 8.69 µg/mL, against promastigotes and caused ultrastructural alterations, including mitochondrial enlargement [36].

The mechanism of action of EOs is still unknown, because the specific cellular targets have not been identified yet. Nevertheless, it is generally related to cell membrane disruption, owing to their lipophilic properties, which ultimately lead to cell lysis [35]. Additionally, oils can interact with the membrane of mitochondria to produce free radicals that oxidize the macromolecules of the parasite, leading to their death (by apoptosis or necrosis) [35].

In order to understand the active compounds responsible for the anti-leishmanial effect, it is useful to compare the global effect of EOs with those of the isolated major compounds in their natural or synthetic state. Essid et al. [37] compared the anti-leishmanial effect of myrtle EOs and their reported standard compounds, such as *α*-pinene, caracole, *β*-caryophyllene, citronellal, geraniol, camphor, and *p*-cymene, showing that the activity of the whole EOs exceeded that of the isolated marketed standards.

## 4. Materials and Methods

### 4.1. Reagents

The cell line used in this study was murine macrophage RAW 264.7 (ATCC, TIB-71). MTT reagent: thiazolyl blue tetrazolium bromide [3-(4, 5-dimethyl thiazol-2-yl 2, 5-diphenyltetrazolium bromide], sodium dodecyl sulphate (SDS), isopropanol. The reagents used for cell culture were obtained from Sigma (St. Louis, MO, USA), Fluka Chemie (Buchs, Switzerland) and Merck (Nottingham, UK).

### 4.2. Plant Material

The studied *Citrus* species are trees belonging to the Rutaceae family. Leaves were collected on 29 September 2018 between 8 a.m. and 10 a.m., Latitude: 37°02′60.00″ N Longitude: 11°00′60.00″ E, from young trees in a private orchard plantation near Dar Chichou (El Hawaria, Tunisia).

The studied species included Lemon (*Citrus limon* (L.)) cultivar “Eureka”; Orange (*Citrus sinensis* (L.) Osbeck) cultivar “Thomson Washington navel”; and clementina (*Citrus clementina* Hort. ex Tan.) cultivar “Cassar’’, purchased from the GOVPF (Nursery plantation Compulsory Group of Winegrowers and Fruit Producers) in Tunisia. For the first two species, the nomenclature is according to “The Plant List Database” (http://www.theplantlist.org (accessed on 4 August 2022)). The nomenclature of the third species is according to Aleza [38].

- The “Eureka” lemon cultivar is an ever-bearing and basically seedless variety. Throughout the year, the tree produces fruits of a medium size with a yellow and smooth peel. The first “Eureka” selection originated from seed in the 19th century in the USA [39].
- The “Thomson Washington Navel” orange is a “Washington Navel” cultivar improved in the USA from bud selections, in the 19th century. Fruits are generally seedless and less colored; the fruit apex is usually protruded, with the famous large open navel. They mature approximately two weeks earlier [40].
- The “Cassar” *Clementina* cultivar is a local clone discovered in the region of La Sokra [38], Tunisia, in the 20th century. The tree canopy port is basically spherical, with an average vigor. Fruit shape is generally flattened at the apex and rounded on the peduncle side, with a medium caliber, thin skin, and easy coat. Fruits are generally seedless but contain seeds when the orchard has other genotypes.

### 4.3. Essential Oil Extraction by Hydro-Distillation

First, 1 kg of fresh leaves of *Citrus limon* was subjected to conventional hydro-distillation using a modified Clevenger-type apparatus for 3 h, according to the European Pharmacopoeia (2004). The obtained essential oils (EOs) were dried using anhydrous NaSO_4_ and stored at 4 °C in the dark. The yield of EOs was assessed three times and expressed in percentage according to the fresh weight.

### 4.4. Solvent-Free Microwave Extraction (SFME)

Three hundred grams of each plant leaf were separately subjected to SFME. The method was carried out in a laboratory microwave oven (NEOS Milestone, Sorisole (BG), Italy). During experiments, the time (30 min), temperature (gradient temperature as a function of time during the experiment; from 28 to 100 °C), and power (500 W) were controlled. The experimental SFME variables were optimized to maximize EO yield. In a typical SFME procedure performed at atmospheric pressure, the fresh plant materials were separately heated using a fixed power of 500 W, without adding any solvent or water. EOs and aromatic water were simply separated using a separating funnel. The collected EOs was dried using NaSO_4_ (anhydrous) and stored at 4 °C.

### 4.5. GC-MS Analysis

Gas chromatography-mass spectrometry (GC-MS) analysis was carried out on a gas chromatograph; an HP 7890 series (II) coupled to an HP 5975 mass spectrometer (Agilent Technologies, Palo Alto, CA, USA) with electron impact ionization (70 eV). An HP-5MS (5% Phenyl Methyl Siloxane) capillary column (30 m × 0.25 mm, 0.25-μm film thickness, Agilent Technologies, Hewlett-Packard, Santa Clara County, CA, USA) was used. The column temperature was programmed as follows: 40 °C for 1 min, 8 °C/min to 100 °C for 5 min, 10 °C/min to 200 °C for 3 min, 12 °C/min to 300 °C for 20 min. The carrier gas was helium, with a flow rate of 0.9 mL/min and a split ratio of 100:1. Scan time and mass range were 1 s and 50–550 *m/z*, respectively.

### 4.6. Identification of EO Components

Identification of volatile oils components was achieved by matching the resulting mass spectra with those reported in the Wiley 9/NIST11 mass spectral library of the GC/MS data system, as well as other published mass spectra. Additionally, the retention index, calculated using a mixture of *n*-alkanes, was compared, regarding those occurring in the literature [41,42,43,44]. The calculation of the percentages of the compounds was established using peak area normalization and without the use of correction factors.

### 4.7. Leishmania Parasite Culture

*Leishmaniamajor* (MHOM/TN/95/GLC94) and *Leishmania infantum* (Lv50) strains isolated from patients were used. Amastigotes were obtained after passage in BALB/c mice footpad and harvested from skin lesions. Promastigotes were cultured in a solid medium at 26 °C, and then progressively adapted to a complete medium, composed as indicated in the cell culture section. With a starting concentration of 3 × 10^6^ parasites/mL, the stationary phase, when parasites are in their infective metacyclic forms, was reached after 6 days of culture.

### 4.8. Anti-Leishmanial Activity

Promastigotes forms of *L.major* (MHOM/TN/95/GLC94) and *L.infantum* (Lv50) were grown in M-199 medium supplemented with 40 mM HEPS, 100 µM adenosine, 0.5 mg/L hemin, 10% heat-inactivated fetal bovine serum (FBS), and 50 gentamycin at 26 °C in a dark environment, under an atmosphere of 5% CO_2_. The experiments were performed with parasites in their logarithmic phase of growth. First, 96-well microtiter plates were placed at 26 °C in an atmosphere of 95% air/5% CO_2_. Then, 200 microlitres of culture medium was placed in each well that contained the maximum concentration of the compound to be tested (C1), with 100 µL in the following (C2–C6 and controls): 2 µL of each extract dissolved in DMSO was added in C1, and a serial dilution was performed in the well. After one hour (1 h), 100 µL of culture medium complemented with 10^7^ promastigotes/mL from a logarithmic phase culture was added to the well. The final volume in each well was 200 µL. The effects of EOs on Leishmania species promastigotes were evaluated by the tetrazolium-dye (MTT reagent) colorimetric method. Promastigotes were treated with plant EOs (Concentration of EOs varies from 0.03 to 1 µg/mL), then microliter plates were centrifuged at 2500× *g* for 10 min and supernatants were removed and replaced with the same volume of 2 mg/mL of MTT freshly dissolved in PBS. Plates were incubated overnight at room temperature and centrifuged at 2500× *g*. Formazan salt formed inside the parasite’s mitochondria was solubilized, by discarding supernatants and adding SDS 20% for 2 h and 30 min at 37 °C in the dark. Absorbance was measured at 540 nm using an ELISA plate reader. The optical density (OD) of each treated sample was compared to those grown without extracts. The amount of color produced is directly proportional to the number of viable cells. The results are expressed as the concentration inhibiting parasite growth by 50% (IC_50_) after a 3-day incubation period. Amphotericin B was used as a positive control.

### 4.9. Cell Viability Assay

The cytotoxic effect of the oil was evaluated using a conventional MTT assay, according to the method described in our last publication [45,46]. This assay is based on the capacity of mitochondrial succinate dehydrogenase from metabolic active viable cells to convert the soluble yellow tetrazolium salt into an insoluble formazan product. After cell incubation, cellular supernatants were removed and replaced with 100 µL of 0.2 mg/mL of MTT reagent dissolved in PBS. Cells were then incubated for 2 h at 37 °C, 5% CO_2_. The formazan resulting from the reduction of MTT reagent was solubilized by adding 25 µL of isopropanol, followed by another incubation time of 20 min at room temperature in the dark. The absorbance was measured at 540 nm using an ELISA plate reader, and cell viability was estimated as the percentage of sample absorbance relative to non-treated cells. Each assay was repeated four times as an independent experiment.

### 4.10. Cell and Leishmania Parasite Treatment

RAW264.7 cells were dispatched at 3 × 10^4^ cells per well in 96-well tissue culture plates and kept for one night before treatment. Promastigotes were washed twice with PBS, counted, and dispatched at 10^7^ parasites/well. Cells and parasites were then incubated for 24 h in the presence of increasing concentrations of various extracts (ranging from 0.03 to 1 µg/mL). Negative controls correspond to cells or parasites cultured in the absence of plant oils.

### 4.11. Cytotoxicity Assay and Selectivity Index

The selectivity index (SI) is a parameter defining the balance between the cytotoxicity and biological activity of the compound, corresponding to the highest active concentration with no toxicity and expressed as the ratio of IC_80_ macrophage to IC_50_ parasite. A SI value higher than 1 was considered effective for parasites and safe for macrophages [20]. 

### 4.12. Statistical Analysis

Log transformed IC_80_ and IC_50_ values for cytotoxic and anti-leishmanial were calculated using regression analysis and expressed as a mean of 80% of cell viability and 50% anti-leishmanial activity, respectively. The statistical significance of the differences between the treated and the control sample means was evaluated using Student’s *t*-test and an ANOVA test, for pair-wise and multiple comparisons, respectively. A *p*-value < 0.05 was considered to imply significance. All computations were performed using Excel software (Microsoft Corporation, 2003, Albuquerque, NM, USA).

## 5. Conclusions

In this study, SFME was applied for the first time to isolate the volatile oils from *Citrus* species leaves *(C. sinensis*, *C. lemon*, and *C. clementina)*. This highlighted the advantages of the SFME method to recover the most oxygenated compounds from the oil. A potent anti-leishmanial activity of lemon oil (HD) was produced on *L. major*, followed by *C. clementina*. The same effect was displayed on *L. infantum* by *C. clementina* oil, followed by HD lemon oil. This effect could be attributed to the major compounds, as well as the synergistic effect of different compounds. These findings could be a starting step for the building up of novel, less toxic, and more effective anti-leishmanial drugs, which could become available for low-income populations.

## Figures and Tables

**Table 1:** Chemical composition (%) of the essential oils isolated from *Citrus* species leaves using GC/MS analysis.

N	Compounds	RI ^a^ *Cal*	RI ^a^ Rep	Class ^b^	% *C. clementina* SFME ^c^	% *C. sinensis* SFME ^c^	% *C. limon* SFME ^c^	% *C. limon* HD ^d^
1	*α*-Pinene	939	932	MH	0.19 ± 0.01	1.15 ± 0.01	0.33 ± 0.01	0.27 ± 0.00
2	Sabinene	975	969	MH	0.55 ± 0.01	1.59 ± 0.01	**14.22 ± 0.03**	0.53 ± 0.01
3	*β*-Pinene	979	974	MH	1.81 ± 0.02	-	0.45 ± 0.04	2.77 ± 0.05
4	Myrcene	990	988	MH	0.32 ± 0.04	-	1.29 ± 0.11	0.46 ± 0.01
5	δ-3-Carene	1011	1008	MH	0.52 ± 0.02	1.1 ± 0.02	4.65 ± 0.01	0.97 ± 0.01
6	*p*-Cymene	1024	1020	MH	0.03 ± 0.14	-	0.32 ± 0.01	0.19 ± 0.01
7	Limonene	1029	1024	MH	14.48 ± 0.01	0.53 ± 0.01	4.34 ± 0.09	**27.09 ± 1.89**
8	(*E*)-*β*-Ocimene	1050	1044	MH	0.83 ± 0.02	-	6.09 ± 0.01	1.14 ± 0.03
9	*γ*-Terpinene	1059	1054	MH	0.03 ± 0.01	-	0.48 ± 0.04	-
10	Terpinolene	1088	1086	MH	0.12 ± 0.16	-	1.95 ± 0.04	0.15 ± 0.00
11	Linalool	1096	1095	MO	1.68 ± 0.16	**25.83 ± 0.32**	**44.21 ± 0.52**	1.59 ± 0.07
12	*cis*-Limonene oxide	1132	1132	MO	0.06 ± 0.01	-	-	0.24 ± 0.02
13	Citronellal	1153	1148	MO	0.47 ± 0.01	2.26 ± 0.05	1.22 ± 0.03	0.48 ± 0.03
14	Terpinen-4-ol	1177	1174	MO	0.1 ± 0.16	-	1.84 ± 0.01	0.23 ± 0.01
15	*α*-Terpineol	1188	1186	MO	0.83 ± 0.03	3.47 ± 0.03	4.71 ± 0.03	0.47 ± 0.02
16	Neral	1238	1235	MO	**26.79 ± 0.01**	**27.52 ± 0.32**	2.74 ± 0.31	22.87 ± 0.55
17	Geraniol	1249	1249	MO	-	-	0.77 ± 0.15	-
18	Geranial	1264	1264	MO	**42.40 ± 0.24**	**23.44 ± 0.19**	2.85 ± 0.15	**30.08 ± 0.75**
19	Neryl acetate	1361	1359	MO	0.89 ± 0.06	0.6 ± 0.02	-	2.14 ± 0.06
20	Geranyl acetate	1381	1379	MO	2.27 ± 0.4	-	1.65 ± 0.07	4.02 ± 0.1
21	(Z)-Caryophyllene	1408	1408	SH	0.24 ± 0.03	0.85 ± 0.03	0.38 ± 0.07	0.65 ± 0.02
22	*α*-*trans*-Bergamotene	1434	1432	SH	0.05 ± 0.01	-	-	-
23	Germacrene D	1485	1484	SH		-	0.04	-
24	(*E*, *E*)-α-Farnensene	1505	1505	SH	0.05 ± 0.01	-	0.12 ± 0.01	-
25	*δ*-Cadinene	1523	1522	SH	0.25 ± 0.01	-	-	-
26	(*E*)-*γ*-Bisabolene	1531	1529	SH	0.15 ± 0.01	-	-	0.14 ± 0.01
27	Caryophyllene oxide	1583	1583	SO	0.14 ± 0.01	-	-	-
28	(2*E*,6*Z*)-Farnesol	1715	1714	SO	0.08 ± 0.01	-	-	-
29	α-Sinensal	1756	1755	SO	-	1.48 ± 0.05	0.6 ± 0.06	0.13 ± 0.01
Total identified (%)					**68.54**	**89.82**	**93.96**	**96.67**

^a^ Linear retention index on HP-5 MS column calculated according to the Van Den Dool and Kratz formula (1963). RI cal: calculated retention index, RI rep: reported retention index; ^b^ Chemical class: MH, monoterpene hydrocarbons; MO, oxygenated monoterpenes; SH, sesquiterpene hydrocarbons; SO, oxygenated sesquiterpenes; ^c^ SFME: solvent-free microwave extraction; ^d^ HD: hydro-distillation. The proportions on EOs exceeding 10% are mentioned in bold. Data are presented as means ± S.D (*n* = 3).

**Table 2:** Antileishmanial and cytotoxic activity of *Citrus* EOs.

EOs	IC_50_ ± SD (µg/mL)		IC_80_ ± SD (µg/mL)	SI	
	GLC94	Lv50	RAW 264.7	GLC94	Lv50
E1	1.13 ± 0.3	0.57 ± 0.09	0.30± 0.39	0.26	0.78
E2	1.03 ± 0.27	0.32 ± 0.18	0.18 ± 0.11	0.17	0.56
E3	5.25 ± 0.56	9.48 ± 0.25	0.16 ± 0.09	0.03	0.016
E4	0.9 ± 0.29	0.52 ± 0.15	3.32 ± 0.24	3.68	6.38
AB	0.80 ± 0.18	0.23 ± 0.13	9.23 ± 0.13	41.95	11.53

E1 (*C. limon*, SFME), E2 (*C. clementina*), E3 (*C. sinensis*), E4 (*C. limon*, HD), GLC94 (*Leishmania major*), Lv50 (*Leishmania infantum*). RAW 264.7 (RAW Macrophages) AB (Amphotericin B), SI (Selectivity index). Data are presented as Mean SD (*n* = 4).

## Data Availability

Data is contained within the article.
